# Role of Regulatory T cells in Atorvastatin Induced Absorption of Chronic Subdural Hematoma in Rats

**DOI:** 10.14336/AD.2018.0926

**Published:** 2019-10-01

**Authors:** Wei Quan, Zhifei Zhang, Pan Li, Qilong Tian, Jinhao Huang, Yu Qian, Chuang Gao, Wanqiang Su, Zengguang Wang, Jianning Zhang, Alex Zacharek, Poornima Venkat, Jieli Chen, Rongcai Jiang

**Affiliations:** ^1^Department of Neurosurgery, General Hospital of Tianjin Medical University, Tianjin, China.; ^2^Tianjin Key Laboratory of Injuries, Variations and Regeneration of Nervous System, Tianjin Neurological Institute, Tianjin, China.; ^3^Department of Neurosurgery, The First Central Hospital of Tianjin, Tianjin, China.; ^4^Department of Neurology, Tianjin Huanhu Hospital, Tianjin, China.; ^5^Department of Neurology, Tangdu Hospital, Baqiao, Shanxi, China.; ^6^Department of Neurosurgery, The First Central Hospital of Baoding City, Lianchi, Baoding, China.; ^7^Department of Neurology, Henry Ford Hospital, Detroit, MI 48202, USA

**Keywords:** chronic subdural hematoma, atorvastatin, inflammation, regulatory T cell, cytokines

## Abstract

Chronic subdural hematoma (CSDH) is a neurological disorder with a substantial recurrence rate. Atorvastatin is an effective drug for treating hyperlipidemia and known to improve neurological outcome after intracerebral hemorrhage. Previous studies have reported that atorvastatin treatment promotes hematoma absorption in CSDH, while the underlying mechanisms remain unclear. In this study, we investigated whether the anti-inflammatory effects of atorvastatin mediate absorption of CSDH. 144 male, Wistar rats (6 months old) were randomly divided into the following groups: 1) sham surgery control, 2) treatment: CSDH + atorvastatin, and 3) vehicle control: CSDH + saline. Atorvastatin or saline was orally administered daily for 19 days after CSDH procedure. A T2WI MRI was used to evaluate CSDH volume changes during the time course of the study. Flow cytometry and immunohistochemical staining were used to measure the number of regulatory T cells (Treg). ELISA was used to measure cytokine level in the hematoma border. Neurological function and cognitive outcome were evaluated using Foot-Fault test and Morris Water Maze test, respectively. When compared to saline treatment, atorvastatin treatment accelerated the absorption of CSDH as indicated by decreased hematoma volume in T2WI MRI data on 14^th^ and 21^st^ day after CSDH (P<0.05). Atorvastatin treatment significantly increased the number of Treg in circulation and hematoma border from 3^rd^ to 21^st^ day after CSDH. Atorvastatin treatment significantly decreased the levels of interleukins (IL-6 and IL-8) and tumor necrosis factor-α (TNF-α), but increased IL-10 level in the hematoma border. Atorvastatin treatment also improved neurological function and cognitive outcome compared to vehicle treated group. Atorvastatin induced anti-inflammatory responses and increased Treg in circulation and brain which may contribute to the accelerated CSDH absorption in rats.

Chronic subdural hematoma (CSDH) is a common neurosurgical disease. CSDH is often encountered in patients of advanced age, post traumatic brain injury, and in patients treated with anticoagulants [1]. A surgical procedure consisting of burr-hole drainage is routinely used to treat CSDH to resolve acute enlargement of the hematoma. However, surgical intervention also bears a recurrence rate as high as 29%, which necessitates additional surgical procedures and increases overall risk and economic burden [2, 3]. CSDH recurrence after burr-hole drainage is not caused by the surgical procedure rather; it is related to local or systemic inflammatory responses or coagulopathy [4].

Vascular dysfunction and inflammation are major mechanisms of CSDH recurrence. Inflammatory factors such as interleukins (IL-6 and IL-8), tumor necrosis factor-α (TNF-α), and inflammatory cells such as monocytes and macrophages promote CSDH recurrence [5-7]. Regulatory T cells (Treg) inhibit the activation of immune response and exert neuroprotective effects after ischemic stroke, and attenuate cerebral inflammation in subarachnoid hemorrhage (SAH) and intracerebral hemorrhage (ICH) 8, 9]. Atorvastatin is a beta-hydroxy-beta-methylglutaryl coenzyme A reductase inhibitor and is used to reduce the synthesis of cholesterol and isoprenoid in patients [10-13]. Atorvastatin is known to play an important role in the regulation of vascular function and anti-inflammatory responses after stroke or brain injury [10-13]. Atorvastatin treatment of stroke can modulate Treg population in peripheral tissue and favor their accumulation in the brain [14]. Atorvastatin increases the frequency and phenotype of circulating Treg even in healthy individuals [15]. In our previous studies, we found that atorvastatin treatment accelerates the absorption of acute subdural hematoma in rats [16, 17]. In our clinical studies, we found that oral administration of atorvastatin is safe and effective in inducing significant improvement with the resolution of CSDH at 8 weeks [18-20]. However, whether atorvastatin decreases CSDH by regulating Treg is not clear.

In the current study, we investigated whether atorvastatin treatment in rats subjected to CSDH model promotes hematoma absorption and improves neurological function and cognitive outcome by regulating Treg and its related anti-inflammatory effects.

## MATERIALS AND METHODS

All experiments were conducted in accordance with the local institutional ethical standards committee on animal experimentation of Tianjin Medical University (Tianjin, China).

### CSDH Model

Male, 6 months old Wistar rats (Institute of Bioengineering, Chinese Academy of Sciences) were subject to CSDH model following previously published procedures with minor modifications noted below [21]. Briefly, rats were anesthetized with 10% chloride hydrate solution (3.0 ml/kg, administered via intraperitoneal injection) and positioned in a stereotaxic frame (Stoelting, USA). An incision was made to the scalp and muscle, exposing the coronal and sagittal suture. At coordinates 2.5 mm caudal from the right coronal suture and 3 mm lateral to the sagittal suture, a small conical burr hole (1 mm in diameter) was drilled with a sphenoid drill (Medtronic, USA). A small hole on the dura was lacerated carefully under the microscope using a small hooked needle (with a diameter of 0.3 mm) such that there was no injury to the cortex. Then, 400 μl of autologous blood was collected from the angular vein of the rat and immediately drawn into a 1 ml germ-free and anticoagulant-free syringe connected to an 18-gauge intravenous catheter with a tapered tip (BD Vialon, USA). The syringe was fixed on the arm of a stereotaxic injector (QSI Quint essential stereotaxic injection No. 53311; Stoelting, USA) and the tip of a catheter was pushed into the conical burr hole to ensure the tapered tip reached the dura opening. The injector was set to an initial flow rate of 30 μl/min for 10 minutes, and then reduced to 10 μl/min for another 10 minutes to inject a total volume of 400 μl into the subdural space. After the injection, the catheter was left in place for 5 minutes before removal and sealing of the hole using bone wax. The incision was then sutured, and rats placed on a warming blanket for recovery. At 48 hours following the first infusion, we performed the second injection of autologous blood. The rats were anesthetized and incised as described above. The bone wax was carefully removed under a microscope with microsurgical forceps and 300 μl blood was drawn from the angular vein and injected into the subdural space at a flow rate of 20 μl/min for 10 minutes, and then at 10 μl/min for another 10 minutes to enlarge the hematoma. Once again, the burr hole was sealed using bone wax and incision sutured. For sham surgery, the rats were subjected to a similar surgical procedure as the CSDH model, but without any blood injection.

### Experimental groups

Rats were randomized to three groups: 1) Sham surgery control; 2) Vehicle control (CSDH + saline) identified as CSDH-nt; 3) Treatment (CSDH + atorvastatin) identified as CSDH-at. Atorvastatin (5 mg/kg) or saline treatment was initiated at 3 days after the first blood injection and administered via oral gavage daily. 2 sets of animals were prepared for sacrifice on day 3, 14 or 21 after CSDH for functional testing, immunohistochemical analysis, flow cytometry and ELISA assay (n=8/group/time point), with a total of 144 rats employed in this study.

### MRI scanning of the rat head

Magnetic resonance imaging (MRI) using a 3.0 T instrument (MAGNETOM Spectra 3.0T, Germany) was employed to verify successful CSDH formation and measure the hematoma volume on days 3, 14, and 21 after first blood infusion. The specific coil (HD 8Ch High Brain Array, GE) was used, and the volume of the CSDH was measured based on the GE MRI T2WI scan with 1mm cuts, based on previous publications [21]. Intracranial hematoma volume was calculated based on T2WI images and by histopathology. The volume tested by MRI was correlated with histopathological staining [21]. A successful CSDH model was identified with an oval-shaped hypointensity between the dura and the frontal-temporal lobe without cortex penetration, and a failed procedure was identified by the presence of cortical contusion, laceration and destroyed structural integrity of cortical surface attributed to the infusion of blood [21]. All of the image slices were collected with the GE workstation software, and hematoma volume was calculated by a 3D sequence of T2WI scans and the software’s automatic volume measurement analysis.

### Brain tissue dissection

On days 3, 14 and 21 after CSDH, rats were sacrificed under deep anesthesia and perfused with PBS followed by 4% paraformaldehyde. A high speed microsaw (Proxxon, Germany) was used to remove the part of skull and brain tissue to keep the integrity of durometer and the capsule of hematoma. The sections were dehydrated and embedded in paraffin. A series of 6 μm sections were cut from the standard paraffin block (through the hematoma area) for immunohistochemical staining.

### Immunohistochemical staining

Different concentration gradients of dimethylbenzene and alcohol were used to remove paraffin and dehydrate the tissue samples. Non-speciﬁc endogenous peroxidase activity was blocked using 3% hydrogen peroxide in methanol for 30 minutes. After permeabilization with 0.1% Triton X-100 for 10 minutes, sections were washed with PBS. The sections were incubated with antibody against FoxP3 (1:50, Abcam, USA) overnight at 4°C. Foxp3 is critical for the development and function of Tregs in mice and humans. FoxP3 is still the only marker for evaluating Tregs that suppress inflammation and is recognized as a more specific marker for Treg than CD4, CD25 and others [22,23]. After washing, tissues were incubated with a biotinylated anti-mouse IgG (1:100, Santa Cruz Biotechnology) for 2 hours at 37°C and then washed and incubated with an avidin peroxidase conjugate solution (1:100, Santa Cruz Biotechnology, USA) for 1 hour. Negative controls were treated as above but without the primary antibody.

### FoxP3 quantification

Brain sections were observed under a light microscope (Leica DM4B microscope (100×)). 5 areas (four corners and one central area) were selected from each brain slide. For each field of view, FoxP3 positive cell numbers were counted using an image-processing and analysis system (ImageJ 1.37 software; NIH, USA). Data were averaged to obtain a single value for each animal and presented as number of positive cells/areas.

### Flow cytometry

To detect the levels of Treg cells in the circulating blood, ?ow cytometric analysis was emplored. On days 3, 14 and 21 after the CSDH, we collected 1 ml of peripheral blood from the angular vein. The blood samples were used to isolate and measure Treg. Using Percoll, the mononuclear cells were centrifuge-separated from blood samples at 2400 rpm for 20 minutes at 25°C. The cloudy layer (mononuclear cells cluster) between the supernate and Percoll were collected and washed three times in PBS, then resuspended in 100 μl of 1% BSA (bovine serum albumin)-PBS solution. CD4^+^CD25^+^FoxP3^+^ triple labeling was employed to identify Treg cells [24]. 100 μl of resuspended mononuclear cells were incubated with 10 μl PE-conjugated CD4 antibody and 10 μl FITC-conjugated CD25 antibody (Miltenyi Biotech, Gladbach, Germany) for 15 minutes in the dark. The cells were washed with 1 ml PBS and centrifuged at 1500 rpm for 10 minutes, then resuspended and treated with 1 ml of 1:3 ﬁxation:permeabilization working solution for 60 minutes. Then, the cells were washed with 2 ml of permeabilization buffer and centrifuged twice at 1500 rpm for 5 minutes. The sample were resuspended with 80 µl of permeabilization buffer and then incubated with 20 μl of APC (allophycocyanin)-conjugated FoxP3 antibody (Miltenyi Biotech, Gladbach, Germany) for 30 minutes in the dark. After the incubation, the samples were washed twice with 2 ml permeabilization buffer. The cells were resuspended with 300 μl PBS and then analyzed using a ?ow cytometer (Becton Dickinson and Company, Franklin Lakes, NJ, USA). Three controls for PE, FITC and APC-conjugated mouse immunoglobulin G, and three single labels as single-channel controls were used.

### Inflammatory factor expression measured by ELISA

The hematoma membrane and the membrane edge tissue were removed from the brain and snap frozen with liquid nitrogen and stored at -80°C until used. Eight samples from each group were obtained. The homogenate was centrifuged at 5000 rpm for 5 minutes and maintained at 4°C, the supernatant fluid was used to measure the levels of IL-6, IL-8, IL-10 and TNF-α, using a commercially available Enzyme Linked Immunosorbent Assays (ELISA) kit (eBioscience, San Diego, CA). All operating procedures were performed according to the manufacturer’s directions.

### Foot-fault test

To evaluate motor instability of forelimbs, foot-fault test was performed on days 3, 14 and 21 after CSDH. The apparatus consisted of using a grid floor with 2 cm×2 cm square vacancies at 5 cm higher than the floor. The movement of each forelimb was considered as 1 step. Totally 100 steps were continuously counted, and the number of foot faults were recorded. The result is expressed as a percentage of foot-fault over the continuous 100 steps [25].

**Figure 1.**
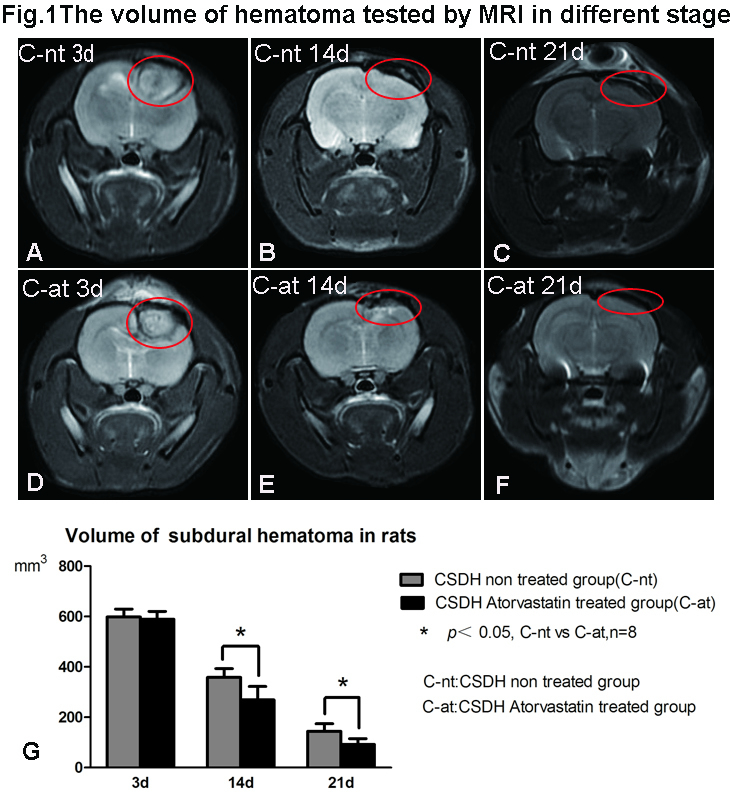
Measurement of hematoma volume at various time points after CSDH using MRI. T2WI sequence MRI scanning shows that atorvastatin treatment decreases hematoma volume from 14 to 21 days after CSDH. (A-C) Representative MRI images of the CSDH saline-treated rats and (D-F) CSDH-atorvastatin treated rats. (G) Hematoma volume quantitative data.

**Figure 2.**
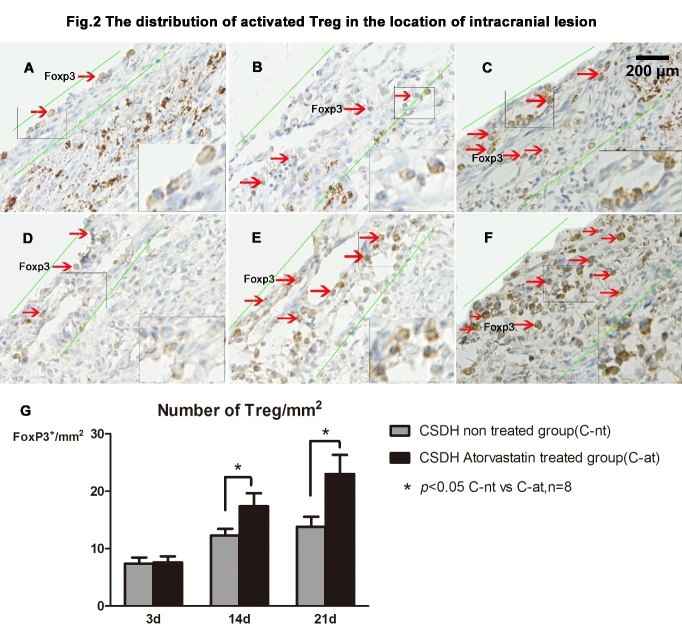
Atorvastatin treatment increases Treg expression in brain tissue of CSDH rats. FoxP3 immunohistochemical staining was used to detect Treg expression in brain tissue. FoxP3+ cells are primarily detected in the vicinity of the intracranial hematoma lesions, and atorvastatin promotes its expression. The dura membrane and its hematoma on days 3, 14 and 21 after CSDH are represented in panels A-C for non-treated CSDH rats, and in panels D-F for atorvastatin treated CSDH rats with quantitative data presented in panel G.

### Morris water maze (MWM) test

To evaluate spatial learning and memory, MWM test was performed for 5 consecutive days i.e. 20-24 days after CSDH. The MWM test is a common method to determine hippocampus based spatial learning and memory. Briefly, a pool was divided into 4 equal quadrants with a platform submerged in the 3^rd^ quadrant. Rats are evaluated on the time required to find the platform while recorded by a video monitor. Rats were tested in 2 sessions per day for 5 days total. Each session included 4 trials and there was a 10 min break between two trials. The escape latency and the swimming route were recorded by the video recording system [21]. The testing apparatus was set up and data collected as described previously [26,27].

### Statistical analysis

Statistical analysis was performed using SPSS 16.0, Data were analyzed for normal distribution. Linear correlation analysis was used for correlation of CSDH lesion volume and Treg expression. A two-way repeated measures ANOVA with a post-hoc Holm-Sidak test was used to test differences between different groups in behavioral outcome measures, immunohistochemical staining, flow cytometry and cytokine array results. A p-value of less than 0.05 was considered as statistically significant and the data are presented as mean±SEM (Standard Error of Mean).

## RESULTS

### Atorvastatin treatment of CSDH increased hematoma absorption

Figure 1 shows representative MRI images and quantification of hematoma volume on days 3, 14 and 21 after CSDH induction. On day 3 after CSDH formation, the subdural hematoma compresses the ipsilateral brain tissue and lateral ventricle, and the midline appears to be shifted. There were no significant differences in hematoma volume between the CSDH-nt and CSDH-at rats on day 3. The volume of hematoma decreased gradually, and atorvastatin treatment significantly accelerated the absorption of the hematoma from 14 to 21 days compared to CSDH-nt rats (P<0.05).

### Atorvastatin increased Treg number in the brain after CSDH

Treg changes within the hematoma and at the boundary of the brain hematoma were assessed using FoxP3 immunostaining. Figure 2 shows that there were no significant differences between the CSDH-nt and CSDH-at groups with respect to FoxP3 expression (marker of Treg) 3 days after CSDH induction, while atorvastatin treatment significantly increased FoxP3 expression by 14 and 21 days after CSDH compared to the saline treated CSDH rats (P<0.05). Furthermore, FoxP3+ Tregs were mainly localized in the vicinity of the intracranial lesions of hematoma, such as on the dura and the neomembrane between the hematoma and cortex surface.

**Figure 3.**
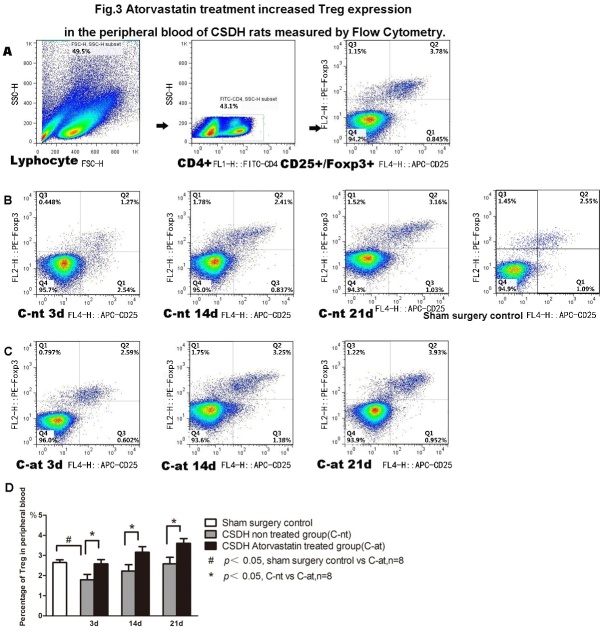
Atorvastatin treatment increases Treg expression in peripheral blood of CSDH rats as measured by flow cytometry. A) Representative dot plots showing the gating strategy. B) Representative dot plots of Treg cells in the sham control and CSDH-non-treated rats. Panel C) Representative dot plots of Treg cells in Atorvastatin treated rats. Quantitative data for Treg in peripheral blood is presented (D).

**Figure 4.**
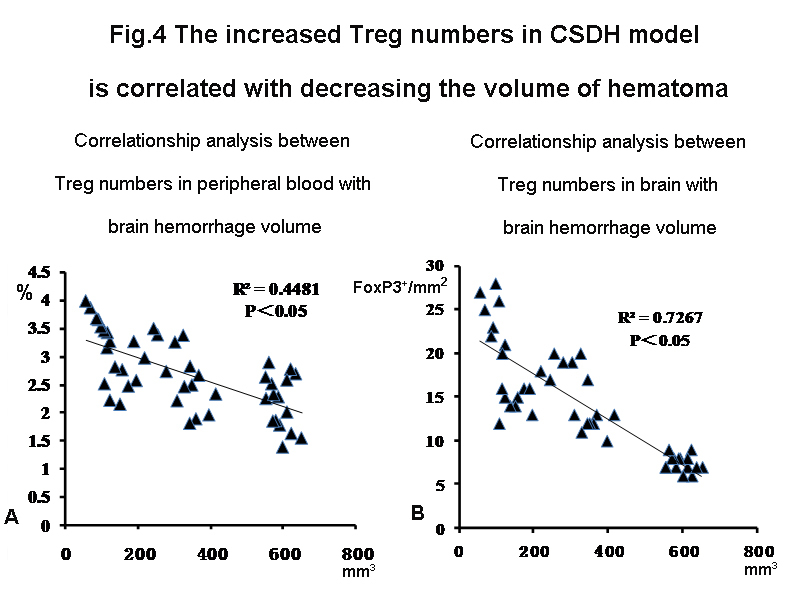
Higher circulating Treg correlates with lower hematoma volume in CSDH rats. Linear correlation analysis indicates that lower hematoma volume after CSDH correlates with Treg increase in peripheral blood (r^2^= 0.448, P<0.05, panel A), and brain (r^2^= 0.727, P<0.05, panel B).

### Atorvastatin treatment increased Treg number in the peripheral blood of CSDH rats

Treg number in the peripheral blood was measured using Flow Cytometry. Figure 3 showed that the number of Tregs in the blood of CSDH rats was significantly decreased compared to the sham control rats. The decreased Treg numbers spontaneously recovered from day 3 to 21 after CSDH (P<0.05). Atorvastatin treatment significantly increased Treg level in blood on days 3, 14 and 21 compared to CSDH-nt rats.

### Treg number in peripheral blood and brain is correlated with decreasing hematoma volume in CSDH rats

To test the relationship between Treg number and CSDH hemotoma volume, linear correlation analysis was employed. Figure 4 showed that the higher Treg number in peripheral blood (r^2^= 0.448, *P*<0.05) as well as brain (r^2^= 0.727, *P*<0.05) significantly correlated with the lower hematoma volume in CSDH rats.

### Atorvastatin treatment after CSDH increased brain IL-10 within the cranial lesion, and decreased brain IL-6, IL-8, and TNF-α expression

**Figure 5.**
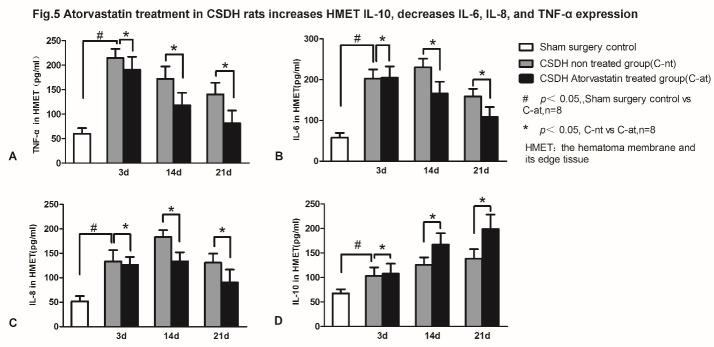
Atorvastatin treatment increases IL-10, and decreases IL-6, IL-8, and TNF-α expression in brain tissue of CSDH rats. Atorvastatin treatment significantly decreases expression of (A) TNF-α, (B) IL-6 and (C) IL-8 while increasing (D) IL-10 expression in the dura and hematoma boundary tissue compared to saline-treated CSDH rats. #P<0.05 for CSDH saline treated group vs. sham control group; *P<0.05 for CSDH atorvastatin treated group vs. CSDH saline treated group.

To determine the nature of soluble inflammatory factors involved in the development of CSDH and the effects of atorvastatin treatment, pro-inflammatory cytokines TNF-α, IL-6, IL-8 and anti-inflammatory IL-10 expression levels were measured in the brain tissue using ELISA. CSDH significantly increases the level of IL-6, IL-8 and TNF-α in the hematoma membrane and its boundary brain tissue on days 3 and 21 after CSDH formation compared to rats subjected to sham surgery. Atorvastatin treatment does not alter inflammatory profile at 3 days after CSDH induction, but significantly decreases IL-6, IL-8 and TNF-α expression on days 14 and 21 compared to CSDH-nt rats. Atorvastatin treatment also increases anti-inflammatory cytokine, IL-10 expression at 3, 14 and 21 days after CSDH as indicated in Figure 5.

### Atorvastatin treatment improves cognitive and neurological function in CSDH rats

**Figure 6.**
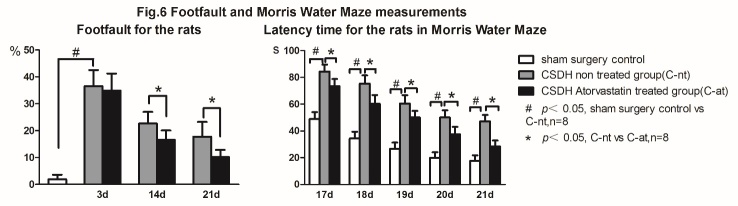
Atorvastatin treatment improves neurological and cognitive outcome in CSDH rats. A) CSDH induces significant neurological impairment compared to sham control rats, and Atorvastatin treatment improves neurological function as indicated by foot-fault test. B) CSDH induces significant cognitive impairment compared to sham control rats, and Atorvastatin treatment improves spatial learning and memory as indicated by Morris water maze test. #P<0.05, CSDH non-treated group vs. sham control group and *P<0.05, CSDH Atorvastatin treated group vs. CSDH-non-treated group.

To test whether atorvastatin treatment improves neurological function and cognitive outcome, we employed foot-fault test and MWM test, respectively. Figure 6 shows that CSDH in rats induces significant neurological deficits indicated by higher number of foot-fault compared to sham surgery control rats. CSDH also induces significant cognitive impairment indicated by higher latency in MWM test compared to sham surgery control rats. Atorvastatin treatment significantly decreases neurological deficits as well as significantly improves cognitive ability compared to CSDH-nt rats as evaluated by foot-fault test and MWM test, respectively.

## DISCUSSION

In this study, we found that the atorvastatin treatment promotes absorption of hematoma after CSDH, significantly improves neurological function and cognitive outcome, as well as decreases inflammatory cytokines in the brain tissue surrounding the hematoma. We report for the first time that atorvastatin increases Treg and decreases inflammatory cytokines both in the circulation and in the brain tissue adjacent to the hematoma following CSDH in rats.

Independent of its lipid-lowering effects, atorvastatin is known to reduce nitric oxide synthase (iNOS) and myeloperoxidase (MPO) expression in the peri-hematoma brain tissue after ICH and decrease peri-hematoma cell death which was associated with improved neurological recovery and anti-inflammatory effects [28, 29]. Atorvastatin promotes absorption of acute subdural hematoma in rats along with a marked inhibition of inflammatory cytokines and a significant increase of pro-angiogenic effects [16]. In addition, our previous studies have found that atorvastatin treatment in patients with CSDH significantly improves neurological functional outcome and effectively reduces brain hemorrhage without any significant complications [18-20, 30]. Consistent with our previous publications, in this study, we found that atorvastatin treatment not only promotes the absorption of CSDH, but also significantly improves neurological function and cognitive outcome after CSDH in rats. Atorvastatin treatment also significantly increases circulating and brain tissue Treg levels and induces anti-inflammatory effects in CSDH rats.

Treg are a specialized lineage of suppressive CD4+ T cells that act as critical negative regulators of inflammation in various biological contexts, and they acquire strongly enhanced inhibitory function when exposed to inflammatory conditions [31, 32]. Treg exert immunosuppressive effects on monocytes/macrophages and likely decrease their transition to a pro-inflammatory state. The imbalance of Treg and CD4+IL-6+ T cells may play a role in the development of some vascular diseases [33]. Based on the chemotactic responses to IL-6 and IL-8 at the lesion site, Treg can migrate to the site of inflammation and down regulate immune reaction [34]. They may potentially reduce the capacity of monocytes and macrophages to secrete pro-inflammatory mediators such as IL-1β, IL-6, IL-8, and TNF-α, and directly generate IL-10, IL-13 and other critical anti-inflammatory cytokines [35].

FoxP3+ Tregs are gradually activated in the border of CSDH, and the increase in Treg number is closely associated with higher hematoma absorption rates during atorvastatin treatment. Tregs are traditionally not present in the central nervous system except in microglia [36]^.^ Therefore, it is likely that atorvastatin treatment activates Treg in the circulation, which then migrate to the local dura and border of the hematoma. Treg have been reported to improve locomotor function in the chronic phase of spinal cord injury via regulation of anti-inflammatory cytokines [37]. In our study, atorvastatin treatment induces the expression of IL-10, which is a key anti-inflammatory factor that is usually elevated along with the activation of Treg. It is likely that atorvastatin induced hematoma absorption is derived from its anti-inflammatory and immunomodulatory effects [38, 39]. Additionally, Treg also regulate angiogenesis which is another important mechanism regulating the prognosis of CSDH [24]. It has been reported that therapeutic intervention of ICH with atorvastatin significantly increases angiogenesis and synaptogenesis in the rim of hematoma [40]. Therefore, Treg may play a powerful role in the general and local immune status after CSDH induction. The activation and accumulation of Treg in both circulation and boundary rim of the hematoma may be a major reason for the inhibition of inflammation and absorption of hematoma following atorvastatin treatment.

### Conclusions

Our study shows that atorvastatin treatment of CSDH in rats promotes the activation of Treg, accelerates hematoma absorption, and improves neurological function and cognitive outcome compared to saline treated CSDH rats. Increasing Treg may play an important role in suppressing inflammatory responses and promoting hematoma absorption, thereby improving functional outcome after CSDH.
